# Comparison of Adverse Kidney Outcomes With Empagliflozin and Linagliptin Use in Patients With Type 2 Diabetic Patients in a Real-World Setting

**DOI:** 10.3389/fphar.2021.781379

**Published:** 2021-12-21

**Authors:** Yueh-Ting Lee, Chien-Ning Hsu, Chung-Ming Fu, Shih-Wei Wang, Chiang-Chi Huang, Lung-Chih Li

**Affiliations:** ^1^ Division of Nephrology, Department of Internal Medicine, Kaohsiung Chang Gung Memorial Hospital and Chang Gung University College of Medicine, Kaohsiung, Taiwan; ^2^ Department of Pharmacy, Kaohsiung Chang Gung Memorial Hospital and Chang Gung University College of Medicine, Kaohsiung, Taiwan; ^3^ School of Pharmacy, Kaohsiung Medical University, Kaohsiung, Taiwan; ^4^ Institute for Translational Research in Biomedicine, Kaohsiung Chang Gung Memorial Hospital and Chang Gung University College of Medicine, Kaohsiung, Taiwan

**Keywords:** acute kidney injury, type 2 diabetes, SGLT2 inhibitor, empaglifiozin, linagliptin

## Abstract

**Background:** To compare the effects of empagliflozin and linagliptin use on kidney outcomes of type 2 diabetes mellitus (T2DM) patients in a real-world setting.

**Methods:** The study involved a propensity score-matched cohort comprising new users of empagliflozin or linagliptin with T2DM between January 1, 2013 and December 31, 2018 from a large healthcare delivery system in Taiwan. Clinical outcomes assessed: acute kidney injury (AKI), post-AKI dialysis, and mortality. Cox proportional hazard model was used to estimate the relative risk of empagliflozin or linagliptin use; a linear mixed model was used to compare the average change in estimated glomerular filtration rate (eGFR) over time.

**Results:** Of the 7,042 individuals, 67 of 3,521 (1.9%) in the empagliflozin group and 144 of 3,521 (4.1%) in the linagliptin group developed AKI during the 2 years follow-up. Patients in the empagliflozin group were at a 40% lower risk of developing AKI compared to those in the linagliptin group (adjusted hazard ratio [aHR], 0.60; 95% confidence interval [CI], 0.45–0.82, *p* = 0.001). Stratified analysis showed that empagliflozin users ≥65 years of age (aHR, 0.70; 95% CI, 0.43–1.13, *p* = 0.148), or with a baseline eGFR <60 ml/min/1.73 m^2^ (aHR, 0.97; 95% CI, 0.57–1.65, *p* = 0.899), or with a baseline glycohemoglobin ≦7% (aHR, 1.01; 95% CI, 0.51–2.00, *p =*0.973) experienced attenuated benefits with respect to AKI risk. A smaller decline in eGFR was observed in empagliflozin users compared to linagliptin users regardless of AKI occurrence (adjusted β = 1.51; 95% CI, 0.30–2.72 ml/min/1.73 m^2^, *p =* 0.014).

**Conclusion:** Empagliflozin users were at a lower risk of developing AKI and exhibited a smaller eGFR decline than linagliptin users. Thus, empagliflozin may be a safer alternative to linagliptin for T2DM patients.

## Introduction

Type 2 diabetes mellitus (T2DM) is a globally recognized critical health issue that generates a heavy economic burden on public health systems (Khan et al., 2020). T2DM has also been identified as an independent risk factor for acute kidney injury (AKI), which is associated with long-term negative effects on the renal system and higher mortality rates among hospitalized patients (Wang et al., 2012; Monseu el al., 2015). Over the past decades, two new classes of glucose-lowering agents (GLAs), sodium-glucose cotransporter 2 inhibitor (SGLT2i) and dipeptidyl peptidase-4 inhibitor (DPP4i), have been introduced and are commonly used in T2DM treatment (Chan and Tang, 2018).

SGLT2i inhibits sodium-glucose cotransporter 2 channels in the renal proximal tubules and enhances glucosuria (Thomas and Cherney, 2018), including empagliflozin, dapagliflozin, and canagliflozin. Empagliflozin exhibits significant cardio-renal benefits, in addition to glycemic control, and is emerging to be an attractive therapeutic option for T2DM (Zinman et al., 2015; Wanner et al., 2016; Wanner et al., 2018). However, the United State Food and Drug Administration has reported on the risk of developing AKI as a result of SGLT2i exposure (U.S. Food and Drug Administration, 2018). Although large randomized clinical trials (RCTs) have shown that empagliflozin preserve long-term kidney function compared to placebo (Wanner et al., 2016; Wanner et al., 2018), there is limited information regarding the impact of empagliflozin on AKI risk and kidney function in the real-world setting (Nadkarni et al., 2017; Ueda et al., 2018; Cahn et al., 2019). Moreover, as a consequence of the highly selective patient recruitment strategy of RCTs, the association between SGLT2i and AKI risk and kidney function deterioration in T2DM patients with chronic kidney disease (CKD) is unknown (Chu et al., 2019).

In contrast, DPP4i is generally considered a safe GLA and causes a decrease in albuminuria, and thus it has neutral or even slightly beneficial effects on kidney (Groop et al., 2013). It is preferred for patients at a high risk of developing AKI, such as elderly patients or patients with CKD (Paolisso et al., 2012; Walker et al., 2017; Bae et al., 2019). Approximately only 5% of linagliptin, a DPP4i, is eliminated by the kidney, and thus despite impaired renal function, it does not require any dose adjustments (McGill et al., 2013). A daily single dose is well-tolerated, and hence, it is largely prescribed for T2DM patients (Montvida et al., 2018). Furthermore, linagliptin has been shown to be associated with lower odds of AKI, making it a better GLA in the management of hyperglycemia (Sutton et al., 2019; American Diabetes Association, 2021). Hence, this study aimed to compare effect of empagliflozin versus linagliptin on adverse kidney outcomes in real-world T2DM patients with different baseline kidney function.

## Methods

### Patient Database

The one-on-one propensity score matched cohort of new users was sourced from the Chang Gung Research Database, a de-identified set of electronic health records from the network of Chang Gung Memorial Hospitals in Taiwan (Shao et al., 2019). The Chang Gung Memorial Hospitals provide approximately 11% of the reimbursed health services under the Taiwan National Health Insurance program (National Health Insurance Administration, 2020). The Chang Gung Research Database contains patient-level data for diagnosis, prescription, procedure, and laboratory test results in the emergency, inpatient and outpatient departments. The Institutional Review Board of Chang Gung Medical Foundation at Taipei, Taiwan approved the current study (permitted #201900902B0).

### Patient Cohort

**FIGURE 1:**
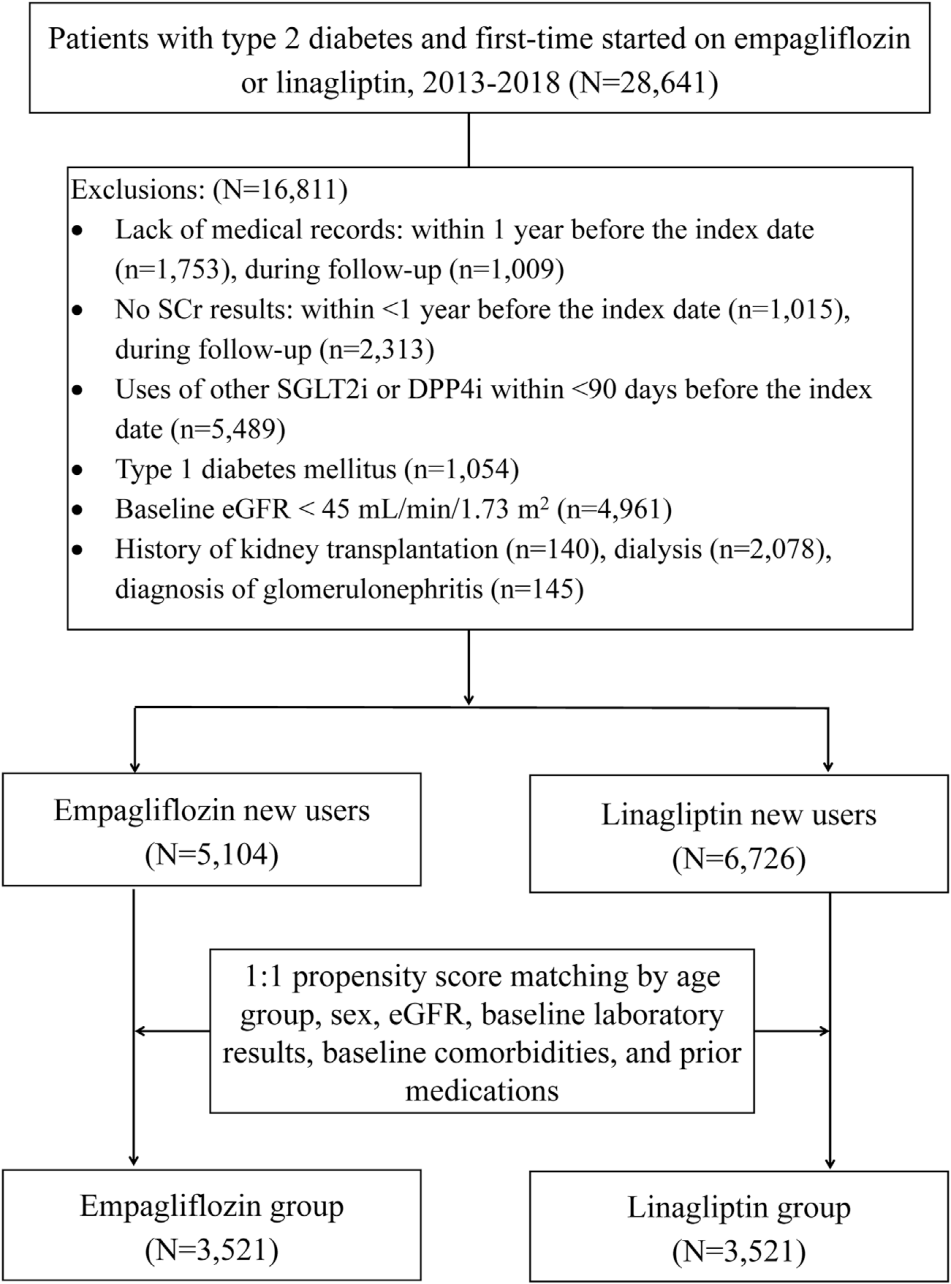
Flowchart and patient selection process. Index date: the date of patient initiated with empagliflozin or linagliptin; DPP4i = dipeptidyl peptidase 4 inhibitors; SGLT2i = sodium-glucose cotransporter 2 inhibitors; SCr = serum creatinine; eGFR = estimated glomerular filtration rate.

The studied cohort consisted of patients with 1) two or more codes of International Classification of Diseases, Nineth and Tenth Revision, Clinical Modification (ICD-9-CM, 250 and ICD-10-CM, E11, respectively) for T2DM with a minimum interval of 28 days from their last an outpatient setting or at least one inpatient claim, 2) at least one prescription of empagliflozin or linagliptin following the date of T2DM diagnosis between January 1, 2013, and December 31, 2018. The expense of GLAs use was covered by Taiwan National Health Insurance program only when patients fulfill the diagnostic criteria of American Diabetes Association guideline (American Diabetes Association, 2020). The first date of empagliflozin and linagliptin use in the outpatient setting served as the index date. To ensure the cohort comprised of new users, patients were excluded due to a lack of medical records pertaining to the 365 days prior to the index date and the use of other SGLT2i (dapagliflozin, canagliflozin) or DPP4i (sitagliptin, vildagliptin, saxagliptin, and alogliptin) within 90 days prior to the index date. To evaluate kidney outcomes, patients were excluded if they had any history of following: type 1 diabetes, baseline eGFR <60 ml/min/1.73 m^2^, kidney transplantation, dialysis, or glomerulonephritis. Further codes and operational definitions were detailed in Figure 1, Supplementary Table S1, and Supplementary Table S2.

### Outcomes

The primary outcome examined in this study was development of AKI during the follow-up period. Secondary outcomes, such as the requirement of dialysis after AKI, in-hospital mortality, and estimated glomerular filtration rate (eGFR) changes from baseline were also compared between the two groups. The first episode of AKI was identified by an increase in serum creatinine (SCr) ≥0.3 mg/dl within 48 h, an increase in SCr to ≥1.5 times baseline within 7 days, or an increase in SCr to ≥4.0 mg/dl as defined by Kidney Disease: Improving Global Outcomes (Kidney Disease: Improving Global Outcomes (KDIGO) Acute Kidney Injury Work Group, 2012). The eGFR was assessed every 3 months using the Modification of Diet in Renal Disease equation [175 × SCr−1.154 × age−0.203 × 0.742 (if female), mL/min/1.73 m^2^] (Levey et al., 1999). The mean eGFR value was an average of the multiple values obtained across the 3 months time window. As empagliflozin has been available since 2016 in the study setting (linagliptin in 2013), patients with a follow-up period of more than 2 years were censored in both treatment groups to minimize attrition bias. We evaluated changes in eGFR from the baseline over the 2 years follow-up. Patients were tracked from the index date until the first episode of AKI, dialysis, in-hospital death, medication discontinuation crossover, or switching or the end of study period (730 days after the index date), whichever came first.

### Covariates

Baseline variables, including patients’ demographic characteristics, laboratory data, comorbid conditions, and prior prescription use of drugs were identified (Table 1). Baseline comorbid conditions, including diabetes complication severity index (Glasheen et al., 2017), were identified using at least two claims of ICD-9/10-CM codes and a minimum interval of 28 days from the last outpatient setting or at least one claim of hospitalization within the 365 days prior to the index date (Supplementary Tables S1, S2). Prior medication uses for ≥28 days was identified in the outpatient setting within the 365 days prior to the index date. Concomitant medication use was also identified in outpatient and inpatient settings within the 3 months prior to the AKI episode or at the end of the follow-up period. Baseline laboratory results and medication use within 3 months before and including the index date are presented in Table 1. We did not conduct multiple imputations for the missing laboratory values.

**TABLE 1:** Baseline patient characteristics before and after propensity score matching.

	Before matching						After matching					
	Empagliflozin (*n* = 5,104)			Linagliptin (*n* = 6,726)			Empagliflozin (*n* = 3,521)			Linagliptin (*n* = 3,521)		
	N	n	(%)	n	(%)	SMD	N	n	(%)	n	(%)	SMD
Age group, years												
<65	7,256	3,690	(72.3)	3,566	(53.0)	**0.603**	4,727	2,349	(66.7)	2,378	(67.5)	**0.018**
≧65	4,574	1,414	(27.7)	3,160	(47.0)	**0.603**	2,315	1,172	(33.3)	1,143	(32.5)	**0.018**
Sex												
Male	7,183	3,125	(61.2)	4,058	(60.3)	**0.090**	4,154	2078	(59.0)	2076	(59.0)	**0.001**
Female	4,647	1979	(38.8)	2,668	(39.7)	**0.090**	2,888	1,443	(41.0)	1,445	(41.0)	**0.001**
Baseline laboratory results												
eGFR (ml/min/1.73 m^2^)												
≧90	4,551	2,376	(46.6)	2,175	(32.3)	**0.625**	3,033	1,533	(43.5)	1,500	(42.6)	**0.019**
60–89.9	4,566	2,140	(41.9)	2,426	(36.1)	**0.469**	2,895	1,433	(40.7)	1,462	(41.5)	**0.017**
45–59.9	2,713	588	(11.5)	2,125	(31.6)	**0.188**	1,114	555	(15.8)	559	(15.9)	**0.003**
HbA1C												
≦7.0	2,140	700	(13.7)	1,449	(21.4)	**0.346**	1,097	563	(15.9)	534	(15.2)	**0.023**
>7.0	9,283	4,346	(85.2)	4,937	(73.4)	**0.453**	5,834	2,901	(82.4)	2,933	(83.3)	**0.024**
Missing	407	58	(1.1)	349	(5.2)	**0.275**	111	57	(1.6)	54	(1.5)	**0.007**
UACR												
No proteinuria(<30 mg/g)	3,492	1826	(35.8)	1,666	(24.8)	**0.413**	2,267	1,144	(32.5)	1,123	(31.9)	**0.060**
Microproteinuria (30–300 mg/g)	2,102	1,066	(20.9)	1,036	(15.4)	**0.167**	1,260	627	(17.8)	633	(17.9)	**0.039**
Macroproteinuria (>300 mg/g)	991	462	(9.1)	529	(7.9)	**0.124**	596	302	(8.6)	294	(8.4)	**0.219**
Missing	5,245	1750	(34.3)	3,495	(52.0)	**0.419**	2,919	1,448	(41.1)	1,471	(41.8)	**0.013**
Individual comorbidity of DCSI												
Retinopathy (0–2)												
0	10,958	4,699	(92.1)	6,259	(93.1)	**0.055**	6,543	3,278	(93.1)	3,265	(92.7)	**0.014**
1	597	304	(5.9)	293	(4.4)	**0.023**	339	168	(4.8)	171	(4.9)	**0.004**
2	275	101	(2.0)	174	(2.6)	**0.123**	160	75	(2.1)	85	(2.4)	**0.019**
Nephropathy (0–2)												
0	8,601	3,719	(72.9)	4,882	(72.6)	**0.578**	5,251	2,625	(74.6)	2,626	(74.6)	**0.001**
1	3,057	1,367	(26.8)	1,690	(25.1)	**0.069**	1748	879	(24.9)	869	(24.7)	**0.007**
2	172	18	(0.4)	154	(2.3)	**0.788**	43	17	(0.5)	26	(0.7)	**0.033**
Neuropathy (0–1)												
0	10,486	4,709	(92.3)	5,777	(85.9)	**0.194**	6,383	3,188	(90.5)	3,195	(90.7)	**0.007**
1	1,344	395	(7.7)	949	(14.1)	**0.194**	659	333	(9.5)	326	(9.3)	**0.007**
Cerebrovascular (0–2)												
0	10,723	4,830	(94.6)	5,893	(87.6)	**0.260**	6,550	3,276	(93.0)	3,274	(93.0)	**0.002**
1	97	27	(0.5)	70	(1.0)	**0.062**	46	23	(0.7)	23	(0.7)	**0.000**
2	1,010	247	(4.8)	763	(11.4)	**0.251**	446	222	(6.3)	224	(6.4)	**0.002**
Cardiovascular (0–2)												
0	8,819	3,670	(71.9)	5,149	(76.6)	**0.030**	5,295	2,642	(75.0)	2,653	(75.4)	**0.007**
1	1,318	618	(12.1)	700	(10.4)	**0.057**	831	416	(11.8)	415	(11.8)	**0.001**
2	1,693	816	(15.9)	877	(13.0)	**0.012**	916	463	(13.2)	453	(12.9)	**0.008**
Peripheral vascular disease (0–2)												
0	11,407	4,986	(97.7)	6,421	(95.5)	**0.149**	6,843	3,418	(97.2)	3,425	(97.3)	**0.012**
1	253	73	(1.4)	180	(2.7)	**0.098**	132	65	(1.9)	67	(1.9)	**0.004**
2	170	45	(0.9)	125	(1.9)	**0.112**	67	38	(1.1)	29	(0.8)	**0.026**
Metabolic (0–2)												
0	11,771	5,096	(99.8)	6,675	(99.2)	**0.342**	7,029	3,515	(99.8)	3,514	(99.8)	**0.007**
2	59	8	(0.2)	51	(0.8)	**0.342**	13	6	(0.2)	7	(0.2)	**0.007**
Baseline comorbid condition												
Hypertension	7652	3270	(64.1)	4832	(65.2)	**0.128**	4383	2190	(62.2)	2193	(62.3)	**0.002**
Hyperlipidemia	7099	3489	(68.4)	3610	(53.7)	**0.359**	4422	2216	(62.9)	2206	(62.7)	**0.006**
Peptic ulcer	1514	521	(10.2)	993	(14.8)	**0.158**	465	228	(6.5)	237	(6.7)	**0.001**
Liver diseases	1879	742	(14.5)	1137	(16.9)	**0.008**	18	9	(0.3)	9	(0.3)	**0.009**
Cancer	1060	259	(5.1)	801	(11.9)	**0.249**	45	23	(0.7)	22	(0.6)	**0.010**
Severe liver diseases	98	9	(0.2)	89	(1.3)	**0.127**	817	409	(11.6)	408	(11.6)	**0.000**
Metastatic cancer	184	24	(0.5)	160	(2.4)	**0.151**	1085	548	(15.6)	537	(15.3)	**0.004**
Prior medication												
Other antidiabetic agents												
Insulins	1448	793	(15.5)	655	(9.7)	**0.076**	813	413	(11.7)	400	(11.4)	**0.012**
Metformin	10977	4979	(97.6)	5998	(89.2)	**0.612**	6807	3402	(96.6)	3405	(96.7)	**0.005**
Sulfonylureas	6986	2967	(58.1)	4019	(59.8)	**0.105**	4133	2063	(58.6)	2070	(58.8)	**0.004**
Acarbose	3913	1724	(33.8)	2189	(32.6)	**0.012**	2265	1131	(32.1)	1134	(32.2)	**0.002**
Thiazolidinediones	2654	1289	(25.3)	1365	(20.3)	**0.109**	1656	832	(23.6)	824	(23.4)	**0.005**
Glucagon-like peptide-1	707	474	(9.3)	233	(3.5)	**0.250**	386	196	(5.6)	190	(5.4)	**0.008**
Meglitinides	1386	420	(8.2)	966	(14.4)	**0.343**	670	337	(9.6)	333	(9.5)	**0.004**
Lipid-lowering agents												
Statins	6791	3527	(69.1)	3264	(48.5)	**0.424**	4290	2145	(60.9)	2145	(60.9)	**0.000**
Fibrates	1179	551	(10.8)	628	(9.3)	**0.046**	756	376	(10.7)	380	(10.8)	**0.004**
Others	298	163	(3.2)	135	(2.0)	**0.078**	197	99	(2.8)	98	(2.8)	**0.002**
Anti-hypertensive medication												
ACEI	805	454	(8.9)	351	(5.2)	**0.126**	450	212	(6.0)	238	(6.8)	**0.030**
ARB	6108	2849	(55.8)	3259	(48.5)	**0.053**	3659	1826	(51.9)	1833	(52.1)	**0.004**
Direct renin inhibitor	43	12	(0.2)	31	(0.5)	**0.043**	20	11	(0.3)	9	(0.3)	**0.011**
Thiazide	274	103	(2.0)	171	(2.5)	**0.047**	154	77	(2.2)	77	(2.2)	**0.000**
Furosemide	776	238	(4.7)	538	(8.0)	**0.332**	362	178	(5.1)	184	(5.2)	**0.008**
Diuretics-potassium sparing	539	237	(4.6)	302	(4.5)	**0.045**	276	139	(3.9)	137	(3.9)	**0.003**
Beta-blockers	3640	1792	(35.1)	1848	(27.5)	**0.072**	2186	1088	(30.9)	1098	(31.2)	**0.006**
Calcium channel blockers	2672	1012	(19.8)	1660	(24.7)	**0.229**	1530	755	(21.4)	775	(22.0)	**0.014**
NSAID	1133	395	(7.7)	738	(11.0)	**0.114**	612	311	(8.8)	301	(8.6)	**0.010**

ACEIs: angiotensin-converting-enzyme inhibitors; ARBs: angiotensin II receptor blockers; DSCI: diabetes complications severity index; eGFR: estimated glomerular filtration rate; NSAID: non steroid anti-inflammatory drug; SMD: standard mean difference; UACR: urine albumin-creatinine ratio. Standardized mean difference (SMD) < 0.10 indicating no significant difference in baseline covariates between two treatment groups.

### Statistical Analysis

Descriptive statistical analysis results of continuous variables were reported as mean and standard deviation or median and interquartile range, and data were summarized as *n* (%) for categorical variables in the study cohort. The incidence rate of AKI (per 1,000 patient-years) was assessed in the overall group and for both treatment groups. Kaplan-Meier analysis with log-rank test was employed to assess the cumulative incidence of AKI over the 2 years follow-up period in the matched cohort and stratified by the baseline eGFR categories (≥ and <60 ml/min/1.73 m^2^).

To mimic a hypothetical random treatment allocation, individuals’ propensity scores for the initiation of empagliflozin or linagliptin treatment were first calculated based on the patient’s age, sex, baseline eGFR, Hemoglobin (Hb)A1C values, medication use, and comorbid conditions (Table 1) using a multivariate logistic regression model. Patients were matched 1:1 based on propensity score using a greedy match algorithm (Parsons, 2004). We evaluated the covariate balance in the study cohort before and after propensity score matching (PSM) by comparing the distribution of propensity scores and calculating a standardized mean difference (SMD) to compare baseline covariates between treatment groups. An SMD of less than 0.10 was considered to be balanced (Austin, 2009).

The Cox proportional hazards regression model was used to estimate the adjusted hazard ratio (aHR) with 95% confidence interval (CI) for the effect of empagliflozin on the risk of AKI, after adjustments for age, sex, baseline laboratory results, comorbid conditions, and concomitant medication use was made (Supplementary Table S3). Changes in eGFR were compared using a linear mixed model with an unstructured covariance matrix of the random effect (Leffondre et al., 2015). Interactions of time and treatment groups, age, sex, and baseline HbA1C were included in the model. The coefficient of the treatment group presents the difference in mean eGFR slope between the empagliflozin and linagliptin groups. All analyses and data management were conducted using SAS (version 9.4; SAS Institute Inc., Cary, NC, United States).

## Results

### Patient Characteristics

The studied cohort comprised 11,830 T2DM patients who were prescribed empagliflozin (*n* = 5,104) or linagliptin (*n* = 6,726) during the study period (Figure 1). Of these patients, 60.7% were male, 38.7% were ≥65 years, and the mean eGFR at baseline was 91.0 ± 27.6 ml/min/1.73 m^2^ (Table 1). HbA1C >7 (78.5%; *n* = 9,283) and a diabetes complication severity index score >0 (58.3%; *n* = 6,857) were common among the patient cohort, as was hypertension (60.7%; *n* = 7,652) and hyperlipidemia (60%; *n* = 7,099). Around 26% (*n* = 3,093) of the cohort had micro- or macro-proteinuria. New users of empagliflozin were younger and had higher mean eGFR at baseline compared with that of new users of linagliptin (*p <* 0.001 for both). Patient baseline characteristics before PSM are provided in Table 1.

The groups were found to be balanced in the PSM analysis (SMD <0.1) (Table 1). The empagliflozin group comprised of 33.3% of patients, while the linagliptin group comprised of 32.5% of the patients that were older than 65 years. The empagliflozin group included 8.6% patients with macroproteinuria versus 8.4% in the linagliptin group.

### Relative Risk of Adverse Kidney Outcomes

There were 211 episodes of AKI, 35 cases of dialysis after AKI, and 36 deaths during the 2 years follow-up period (Table 2). The incidence rate of AKI was 14.3 per 1,000 person-years in the empagliflozin group and 27.5 per 1,000 person-years in the linagliptin group. Patients who had been prescribed linagliptin exhibited a higher rate of stage 3 AKI (1.3 vs. 0.5%), dialysis after AKI (0.7 vs. 0.3%), and death with AKI (0.9 vs. 0.1%) than that among empagliflozin users.

**TABLE 2:** Study outcomes by the treatment groups in the matched cohort.

	Overall	Empagliflozin		Linagliptin		*p*-value
	(*n* = 7,042)	(*n* = 3,521)		(*n* = 3,521)		
AKI, *n* (%)	211	67	(1.9)	144	(4.1)	<0.001
Incidence rate (per 1,000 person-years)		14.3		27.5	
AKI stage, *n* (%)						
1	128	42	(1.2)	86	(2.4)	
2	20	7	(0.2)	13	(0.4)	
3	63	18	(0.5)	45	(1.3)	
Dialysis-acquiring AKI, *n* (%)	35	9	(0.3)	26	(0.7)	0.004
Deaths with AKI, *n* (%)	36	5	(0.1)	31	(0.9)	0.001
Deaths for any cause, *n* (%)	89	23	(0.7)	66	(1.9)	<0.001
eGFR decline changes from baseline (ml/min/1.73 m^2^ per year)						
Median (25th, 75th)		2.4	(−3.9, 9.9)	2.8	(−2.9, 9.6)	0.058

AKI: acute kidney injury; eGFR: estimated glomerular filtration rate. Standardized mean difference (SMD) < 0.10 indicating no significant difference in baseline covariates between two treatment groups.

**FIGURE 2:**
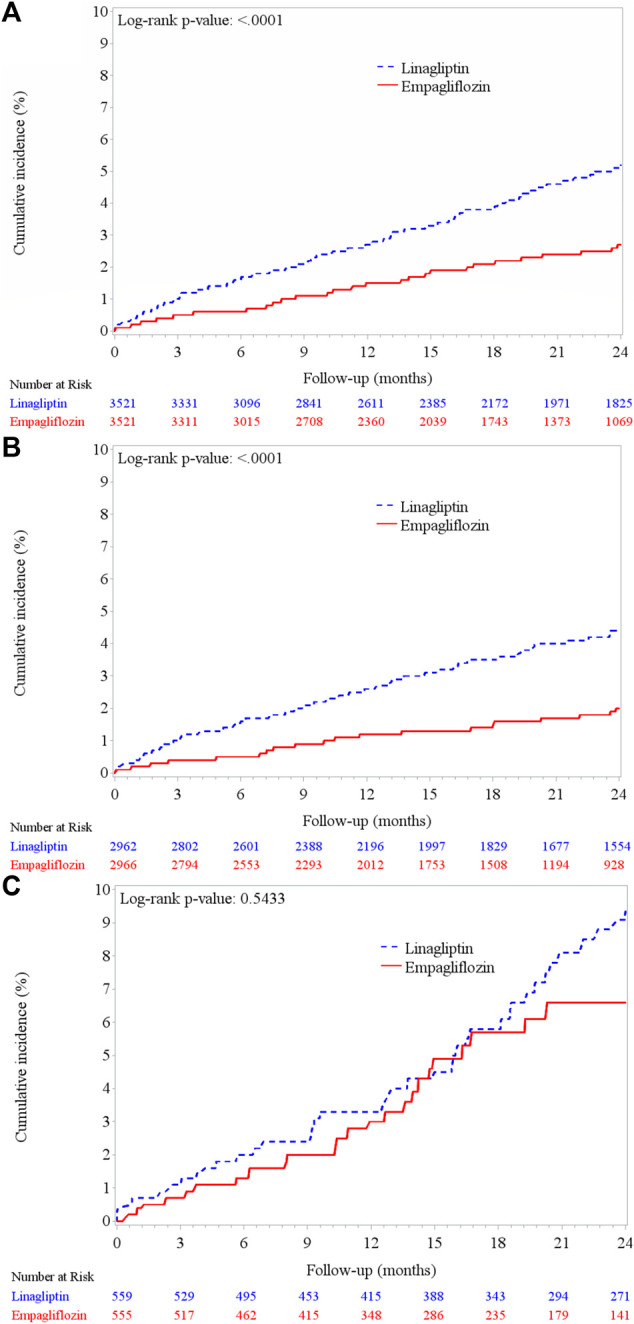
Cumulative incidence of acute kidney injury between empagliflozin and linagliptin groups. **(A)** PSM cohort (*n* = 7,042) **(B)** patients with baseline eGFR ≥60 ml/min/1.73 m^2^ (*n* = 5,928) **(C)** patients with baseline eGFR <60 ml/min/1.73 m^2^ (*n* = 1,114).

**FIGURE 3:**
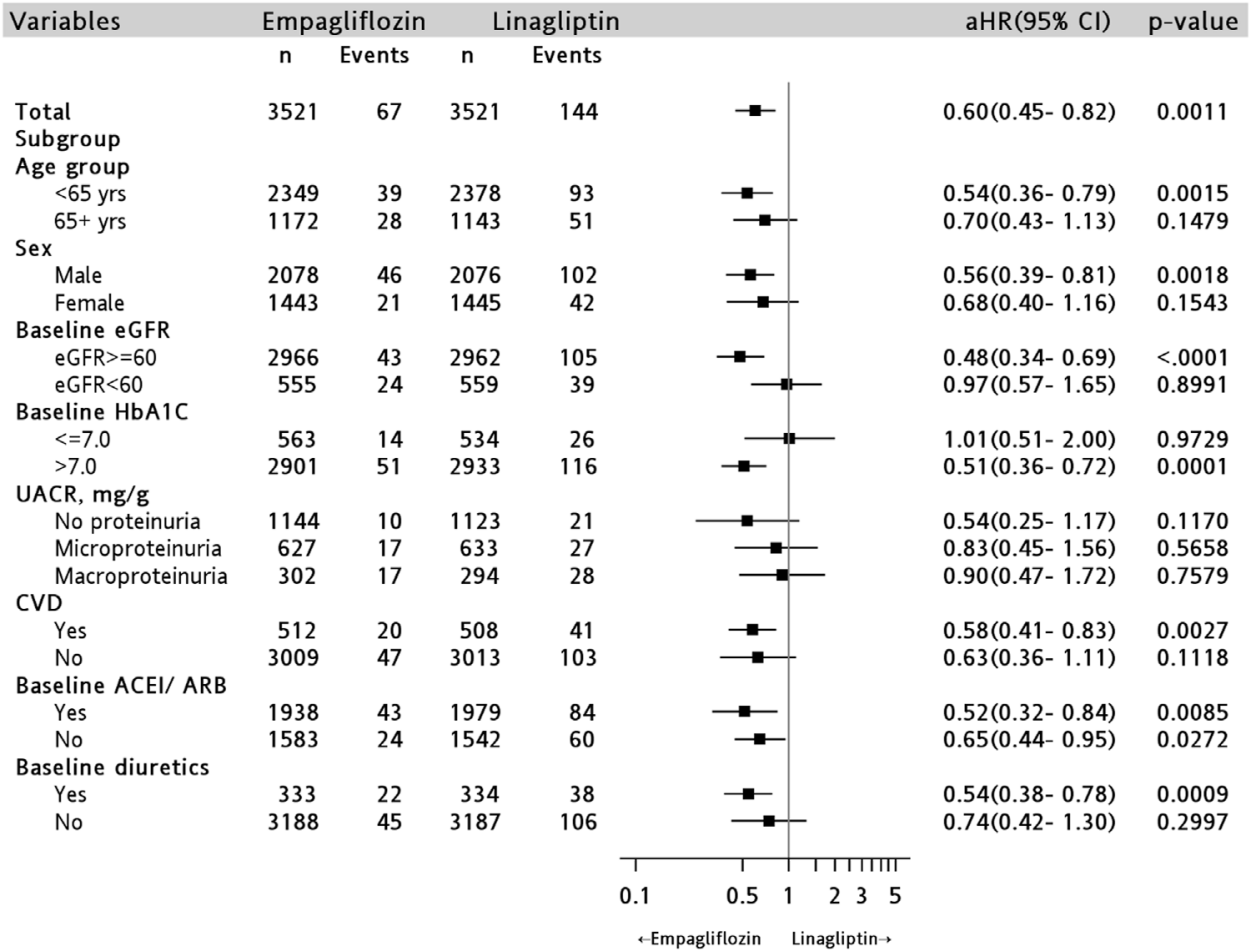
Risk of acute kidney injury comparing empagliflozin with linagliptin groups in the PSM cohort and subgroups. AHR = adjusted hazard ratio by patient age, sex, baseline comorbid, laboratory results and baseline medication users; UACR = urine albumin creatinine ratio; CVD = cardiovascular disease; ACEI/ARB = angiotensin-converting enzyme inhibitors/angiotensin receptor blockers.

The cumulative incidence of AKI among empagliflozin users was lower than that among linagliptin users (log-rank test, *p* < 0.001) (Figure 2A). Similar results were observed for the baseline eGFR ≥60 ml/min/1.73 m^2^ subgroup (Figure 2B), but not in the baseline eGFR <60 ml/min/1.73 m^2^ subgroup (Figure 2C; log-rank test, *p* = 0.54). Empagliflozin was independently associated with a 40% lower risk of AKI compared with that seen among linagliptin users (aHR, 0.60; 95% CI, 0.45–0.82; *p* = 0.001) (Figure 3 and Supplementary Table S3).

In the stratified analysis, we found that among patients with a higher severity of diabetes (HbA1C >7), empagliflozin users were found to be at a relatively lower risk of AKI compared to that of linagliptin users (aHR, 0.51; 95% CI, 0.36–0.72, *p* = 0.0001); this was not seen among patients with HbA1C ≤7 (aHR, 1.01; 95% CI, 0.51–2.00, *p* = 0.97) (Figure 3). Empagliflozin users have also significant lower risks of AKI in male (aHR, 0.56; 95% CI, 0.39–0.81, *p* = 0.002) and age <65 years old (aHR, 0.54; 95% CI, 0.36–0.79, *p* = 0.002) compared to those of linagliptin users (Figure 3).

### Trends in eGFR

The eGFR values declined gradually in both treatment groups over the 2 years observation period (Supplementary Figure S1). The eGFR reduction (baseline minus the last eGFR measure per year) was similar between the empagliflozin and linagliptin (−2.39 vs. −2.79 ml/min/1.73 m^2^ per year) groups without adjusting for AKI incidence (*p =* 0.058) (Table 2, Supplementary Figure S1A). Empagliflozin significantly slowed eGFR decline (adjusted β = 1.51; 95% CI, 0.30–2.72 ml/min/1.73 m^2^, *p =* 0.014) after adjusting for age, sex, baseline HbA1C, and AKI event (Supplementary Table S4).

## Discussion

The results of the 7,042 matched pairs of empagliflozin and linagliptin new user cohort study suggested that empagliflozin was associated with a lower risk of AKI during clinical management of T2DM. Compared with linagliptin, renoprotective effects of empagliflozin against AKI was greater in patients aged <65 years, with poor blood sugar control (HbA1C >7%), and baseline eGFR ≥60 ml/min/1.73 m^2^. Regardless of AKI development, a smaller decline in eGFR was noted in the empagliflozin group compared to that in the linagliptin group.

AKI is recognized as a distinct risk factor for CKD progression and mortality. Timely identification of AKI development is a crucial strategy for CKD prevention in T2DM patients (Xu et al., 2020). To the best of our knowledge, the current study is the first report to compare the risk of developing AKI between new users of empagliflozin and linagliptin among T2DM patients in a real-world setting. Our study demonstrates that empagliflozin is associated with a lower incidence of AKI, and this in line with the findings of beneficial kidney outcomes reported in previous large clinical trials (Zinman et al., 2015; Neal et al., 2017; Wiviott et al., 2019). A meta-analysis of RCTs showed that SGLT2i was significantly associated with a lower risk of developing AKI than placebo (odds ratio: 0.76, 95% CI: 0.66–0.88) (Zhao et al., 2020). In an analysis of T2DM patients in American populations, Nadkarni et al. demonstrated that the risk of developing AKI did not increase among SGLT2i compared to other GLA users (Nadkarni et al., 2017). The analyses carried out in our study were robust since SCr levels changes were used as indicators of AKI, instead of clinical opinion, which may lead to underestimation of AKI development.

Our results revealed that empagliflozin is associated with lower risk of developing AKI in patients with a baseline eGFR ≥60 ml/min/1.73 m^2^ (aHR, 0.48, *p <* 0.001). Previous RCTs have shown no difference in the development of AKI with empagliflozin use compared to that of placebo use among T2DM patients with baseline eGFR between 45–60 ml/min/1.73 m^3^ (Zinman et al., 2015; Wanner et al., 2016). Despite the absence of a significant difference in the risk of AKI between empagliflozin and placebo use among patients with GFR<60 ml/min/1.73 m^2^, there seems to be a decline in AKI in empagliflozin users after 20 months of follow-up (Figure 2C). A long-term follow-up of intervention study is warranted to ensure effect of SGLT2i on CKD progression.

A growing body of literature provides evidence regarding the association of hyperglycemia with the risk of AKI (Kim et al., 2019; Xu et al., 2020). A recent clinical trial involving Asian T2DM patients who took premixed insulin and exhibited inadequate glycemic control reported that the use of empagliflozin aided in better sugar reduction than the use of linagliptin during the 24 months of the treatment regimen (Liu et al., 2021). A pooled analysis comparing the effects of SGLT2i and DPP4i use among T2DM patients with suboptimal sugar control with insulin injection also found that SGLT2i achieved greater reduction of HbA1C (−0.24%, 95% CI: −0.43 to 0.05%) compared to that observed with DPP4i (Min et al., 2017). In addition to glycemic control, SGLT2i was also reported to lower blood pressure, decrease body weight, and mitigate diabetic glomerular hyperfiltration, which are all risks of AKI (Bolinder et al., 2012; Thomson et al., 2012; Bolinder et al., 2014; Monami et al., 2014). Therefore, the lower risk of developing AKI among T2DM patients with inadequate sugar control may be attributed to the pleiotropic effects of empagliflozin compared to that of linagliptin (Terami et al., 2014; Shigiyama et al., 2017; Osonoi et al., 2018). The study results in a subset of patients with A1C >7% may shed light on these concepts and suggested that early use of empagliflozin, especially in patients with poor glycemic control, may result in a lower incidence of AKI.

Our results showed that empagliflozin is more beneficial against AKI than linagliptin in patients under 65 years of age. Moreover, older patients showed a trend of less, yet insignificant, AKI events among new users of empagliflozin compared with that of linagliptin (aHR, 0.70, *p* = 0.15). Recently Iskander et al. conducted a large retrospective cohort study and found that patients younger than 80 years who were new users of SGLT2i experienced lesser number of AKI events than those of DPP4i (Iskander et al., 2020). These study findings highlighted the importance of risk assessment of AKI in the elderly population as they are vulnerable to nephrotoxic medications and co-morbid disease that may increase risk of adverse kidney function (Abaterusso et al., 2008).

Although female was once regarded to confer a higher risk of AKI (Khwaja, 2012), recent studies have reported a higher rate of AKI in male rather than female (Cronin et al., 2015; Loutradis et al., 2021). Our data showed a lower rate of AKI in female than that in male (2.18 vs. 3.56%). Comparing with linagliptin, empagliflozin reduced AKI risk in both male and female patients, but it did not reach statistical significance in the female subgroup (aHR, 0.68, *p* = 0.15). In EMPA-REG OUTCOME trial, empagliflozin versus placebo showed no heterogeneity between male and female (*p* for interaction = 0.85) patients on the composite kidney outcome (Wanner et al., 2016). Further verification is necessary to understand whether sex modifies the effect of SGLT2i on AKI protection. In addition, clinical trial data suggest that SGLT2i can cause body weight loss (Brown et al., 2019), further research can focus on whether body weight or body mass index variability modifies relative effect of empagliflozin on AKI prevention and prognosis in diabetic patients with obesity (Sabaz et al., 2021).

A substantial body of evidence presented by clinical trials and observational studies show a similar retardation of progressive kidney function with SGLT2i use among T2DM populations (Wanner et al., 2016; Wanner et al., 2018; Kanduri et al., 2020; Tuttle et al., 2021). The present study results further demonstrated a biphasic association, shaped like a “check-mark” sign (√) between empagliflozin use and eGFR changes over time, whereas a progressively unitary trajectory of eGFR decrease was observed in linagliptin use. The biphasic eGFR trajectory of empagliflozin use, with or without AKI occurrence, implicated reduced kidney hyperfiltration in the initial 3 months of treatment with SGLT2is, supporting the hypothesis that the acute initial eGFR decline is indicative of a hemodynamic reduction of intraglomerular pressure which endures kidney preservation (Kanduri et al., 2020; Tuttle et al., 2021).

Although our study included a large-scale T2DM population from routine clinical setting, the data should be interpreted with caution owing to certain limitations. First, like other retrospective studies, residual confounding factors that could affect the treatment effect on kidney outcome, including body mass index, smoking, volume status, exposure to sepsis, and major surgeries, were not included in the analyses. Second, only short-term kidney outcomes were evaluated in empagliflozin and linagliptin new users; real-world studies with a longer follow-up period are needed to examine the long-term kidney effect of SGLT2i use. Lastly, treatment pattern and monitoring schedule may not be generalizable to populations of T2DM patients in different healthcare system.

In conclusion, the use of empagliflozin versus linagliptin showed a reduced risk of AKI and slowed eGFR decline in a real-world T2DM population during a 24 months follow-up. In certain conditions, including a baseline eGFR of more than 60 ml/min/1.73 m^2^, HbA1C >7%, or young age (<65 years), empagliflozin is the preferred choice for treatment of T2DM with respect to the renoprotection.

## Data Availability

The original contributions presented in the study are included in the article/Supplementary Material, further inquiries can be directed to the corresponding author.

## Abbreviations

aHR, adjusted hazard ratio; AKI, acute kidney injury; CI, confidence interval; CKD, chronic kidney disease; DPP4i, dipeptidyl peptidase-4 inhibitor; eGFR, estimated glomerular filtration rate; Hb, Hemoglobin; PSM, propensity score matching; RCT, randomized clinical trials; SCr, serum creatinine; SGLT2i, sodium-glucose cotransporter 2 inhibitor; SMD, standardized mean difference; T2DM, type 2 diabetes mellitus
