# Supplementary material for: Comparison of Adverse Kidney Outcomes With Empagliflozin and Linagliptin Use in Patients With Type 2 Diabetic Patients in a Real-World Setting
Source: Front Pharmacol. 2021 Dec 21;12:781379. doi: 10.3389/fphar.2021.781379 (PMC8724779; doi:10.3389/fphar.2021.781379)

**Comparison of Adverse Kidney Outcomes with Empagliflozin and Linagliptin Use in Patients with Type 2 Diabetic Patients in a Real-world Setting**

**SUPPLEMENTAL MATERIALS**

SUPPLEMENTAL TABLE 1. Codes for inclusion/exclusion criteria, baseline comorbid conditions and medication use

SUPPLEMENTAL TABLE 2. Diabetes complications severity index

SUPPLEMENTAL TABLE 3. Factors associated with acute kidney injury

SUPPLEMENTAL TABLE 4. Linear mixed model for assessing the decline in estimated glomerular filtration rate (eGFR)

SUPPLEMENTAL FIGURE 1. Estimated glomerular filtration rate (eGFR) trajectory of empagliflozin and linagliptin users

**SUPPLEMENTAL TABLE 1. Codes for inclusion/exclusion criteria, baseline comorbid conditions and medication uses.**

| **Inclusion criteria:** | |
| --- | --- |
| Type 2 diabetes mellitus | ICD-9-CM: 250 ICD-10-CM: E11 |
| **Medication** | **Anatomical Therapeutic Chemical (ATC) code** |
| Empagliflozin | A10BK03 |
| Linagliptin | A10BH05 |
| **Exclusion criteria:** | |
| Type 1 diabetes mellitus | ICD-9-CM: 250.x1, 250.x3 ICD-10-CM: E10 |
| Kidney transplantation | ICD-9-CM: V42.0 ICD-10-CM: Z94.0 |
| Dialysis | Hemodialysis: M24-003,M24-010,M24-013F,M24-039,M24-040,M24-041, M24-042 , M24-043, M24-045 , M24-046 , M24-047 , M24-048 , M24-049 , M24-050 , M24-051 ,M24-052,M24-053,M24-054,M24-055,M24-056,M24-057 Peritoneal dialysis: 49-444, 58002C, E80-037, M24-001, M24-002, M24-053, M24-054, M24-055,M24-056,M24-057,M24-064,S41-050 |
| Glomerulonephritis | ICD-9-CM: 583.9, 581.3, 582.1, 583.1, 582.2  ICD-10-CM: N02.8, N05.0, N04.1, N03.2, N04.2, N05.2 |
| **Medication** | **Anatomical Therapeutic Chemical (ATC) code** |
| Other SGL2i  (dapagliflozin, canagliflozin) | A10BK01, A10BK02 |
| Other DPP4i  (sitagliptin,vildagliptin,saxagliptin,alogliptin) | A10BH01, A10BH02, A10BH03, A10BH04 |
| **Baseline Comorbidity** | |
| Hypertension | ICD-9-CM: 401-405 ICD-10-CM: I10~I13,I15, N26.2 |
| Hyperlipidemia | ICD-9-CM: 272 ICD-10-CM: E75.2, E75.3, E75.5, E75.6, E77, E78, E88.1, E88.2, E88.89 |
| Peptic ulcer | ICD-9-CM: 272 ICD-10-CM: K25, K26, K27, K28 |
| Liver diseases | ICD-9-CM: 5712, 5714, 5715, 5716 ICD-10-CM: K702, K703, K73, K717, K740, K742, K746, K743, K744, K745 |
| Cancer | ICD-9-CM: 140-208 ICD-10-CM: Cxxx, z5112 |
| Severe liver diseases | ICD-9-CM: 5722, 5723, 5724, 5728 ICD-10-CM: K729, K766, K767, K721 |
| Metastatic cancer | ICD-9-CM: 196, 197, 198, 1990, 1991 ICD-10-CM: C77, C78, C79, C80 |
| **Baseline medications** | **Anatomical Therapeutic Chemical (ATC) code** |
| **Other antidiabetic agents** |  |
| Insulins | A10A |
| Metformin | A10BA02, A10BD |
| Sulfonylureas | A10BB, A10BD02 |
| Acarbose | A10BF |
| Thiazolidinediones (TZD) | A10BG |
| Glucagon-like peptide-1 (GLP-1) | A10BJ |
| Meglitinides | A10BX |
| **Lipid-lowering agents** |  |
| Statins | C10AA,C10BA,C10BX |
| Fibrates | C10AB, C10BA |
| Others | C10AC, C10AX |
| **Anti-hypertensive medication** |  |
| ACEI | C09A, C09B |
| ARB | C09C, C09D |
| Direct renin inhibitor | C09X |
| Diuretics | C03 |
| Thiazide | C03A |
| Furosemide | C03CA01 |
| Diuretics-potassium sparing | C03D, C03E |
| Beta-blockers | C07 |
| Calcium channel blockers | C08 |
| **NSAIDs** | M01AA, M01AX, M01AB, M01AC, M01AE, M01AG, M01AH |
| **Concomitant medications** | **Anatomical Therapeutic Chemical (ATC) code** |
| NSAIDs | M01AA, M01AX, M01AB, M01AC, M01AE, M01AG, M01AH |
| Antibacterial agents for system use | J01G, J01CA, J01CE, J01CF, J01CR, J01E, J02AC01, J01XA01, J01XA02, J01AA07 |
| Diuretics | C03 |
| Thiazide | C03A |
| Furosemide | C03CA01 |
| Diuretics-potassium sparing | C03D, C03E |
| ACEIs | C09A, C09B |
| ARBs | C09C, C09D |
| Immunosuppressants | L04AD, L01BA01 |
| Antiviral agents for system use | J05AB01, J05AB06, J05AB14 |
| Contrast media | V08A,V08B, V08CA |
| ACEIs: Angiotensin-converting-enzyme inhibitors; ARBs: Angiotensin II receptor blockers; DPP4i, dipeptidyl peptidase-4 inhibitor ;NSAID: non steroid anti-inflammatory drug ; SGLT2i, sodium-glucose cotransporter 2 inhibitor | |

| **SUPPLEMENTAL TABLE 2. Diabetes Complications Severity Index. (DCSI)** | | | | | |
| --- | --- | --- | --- | --- | --- |
| **Complication** |  | **ICD-9 Code  (New DCSI) Revised** | **ICD-10 Code (New DCSI) Revised** | **Score**  **:1** | **Score**  **:2** |
| **Retinopathy (0-2)** | Diabetic ophthalmologic disease | x | E08, E09 | • |  |
|  | Background retinopathy | 362.01 | E11.3x, excluding E11.34x, E11.35x | • |  |
|  | Other retinopathy | 362.1 | H35.0x | • |  |
|  | Retinal edema | 362.81-362.83 | H35.6x H35.8x H35.9 | • |  |
|  | CSME | 362.53 | H35.35x | • |  |
|  | Other retinal disorders | 362.81, 362.82 | H35.6x H35.8x H35.9 | • |  |
|  | Proliferative retinopathy | 362.02 | E11.34x E11.35x |  | •• |
|  | Retinal detachment | 361.x | H33.x |  | •• |
|  | Blindness | 369.x | H54.x |  | •• |
|  | Vitreous hemorrhage | 379.23 | H43.1x |  | •• |
| **Nephropathy (0-2)** | Diabetic nephropathy | 250.4, 249.4x | E08, E09, E10, E11, E13 Relevant Subcodes E**.21, E**.22, E**.29 | • |  |
|  | Acute glomerulonephritis | 580 | N00.x | • |  |
|  | Nephrotic syndrome, Hypertension, nephrosis | 581.x | N04.x | • |  |
|  | Chronic glomerulonephritis | 582 | N03.x | • |  |
|  | Nephritis/nephropathy | 583 | N05.x | • |  |
|  | CKD, Stage 1-Stage 3, CKD unspecified | 585.1, 585.2, 585.3, 585.9 | N18.1, N18.2, N18.3, N18.9 | • |  |
|  | CKD, Stage 4-Stage 5, ESRD | 585.4, 585.5, 585.6 | N18.4, N18.5, N18.6 |  | •• |
|  | Renal failure NOS | 586, 593.9 | N19 |  | •• |
|  | Urine protein ≥30 mg/g of creatinine, or (+) dipstick protein or serum creatinine ≥1.5 mg/Dl | UACR, SCr |  | • |  |
|  | Serum creatinine >2.0 mg/dL | SCr |  |  | •• |
|  | eGFR ≤29 mL/min2 (CKD, Stages 4-5) | eGFR |  |  | •• |
| **Neuropathy (0-1)** | Diabetic neuropathy | 357.2, 250.6, 249.6 | E08, E09, E10, E11, E13 Relevant Subcodes E**.4x | • |  |
|  | Cranial nerve palsy | 951.0, 951.1, 951.3 | S04.x | • |  |
|  | Mononeuritis of upper limb and mononeuritis multiplex | 354.x | G56.x | • |  |
|  | Mononeuritis of lower limb and unspecified site | 355.x | G57.x | • |  |
|  | Unspecified hereditary and idiopathic peripheral neuropathy | 356.9 | G60.9 | • |  |
|  | Myasthenic syndromes in diseases classified elsewhere | 358.1 | G73.3 | • |  |
|  | Carotid sinus syncope |  | G90.01 | • |  |
|  | Paralytic strabismus |  | H49.x | • |  |
|  | Charcot’s arthropathy | 713.5 | M14.6x | • |  |
|  | Neurogenic bladder | 596.54 | N31.9 | • |  |
|  | Autonomic neuropathy | 337.0x | G90.09 | • |  |
|  | Peripheral autonomic neuropathy in disorders classified elsewhere | 337.1x | G90.8, G90.9, G99.0 | • |  |
|  | Gastroparesis/diarrhea | 564.5, 536.3 | K31.84, K59.1 | • |  |
|  | Orthostatic hypotension | 458.0 | I95.1 | • |  |
| **Cerebrovascular (0-2)** | TIA | 435 | G45.x | • |  |
|  | Stroke | 431, 433, 434, 436 | I61.x, I63.x, I65.x, I66.x, I67.81 |  | •• |
| **Cardiovascular (0-2)** | Atherosclerosis | 440.x, excluding 440.23 & 440.24 | I70.x, excluding I70.25 & I70.26x | • |  |
|  | Other IHD | 411 | I24.x | • |  |
|  | Angina pectoris | 413 | I20.x | • |  |
|  | Other chronic IHD | 414 | I25.x,excluding I25.2 | • |  |
|  | Other ASCVD | 429.2 | I25.10 | • |  |
|  | Myocardial infarction | 410 | I21.x, I22.x, I23.x |  | •• |
|  | Old myocardial infarction | 412 | I25.2 |  | •• |
|  | Ventricular fibrillation, arrest | 427.3 | I48.x |  | •• |
|  | Atrial fibrillation, arrest | 427.5 | I46.x |  | •• |
|  | Paroxysmal ventricular tachycardia | 427.1 | I47.x |  | •• |
|  | Ventricular fibrillation and flutter | 427.4 | I49.x |  | •• |
|  | Heart failure | 428 | I50.x |  | •• |
|  | Atherosclerosis, severe | 440.23, 440.24 | I70.25 170.26x |  | •• |
|  | Aortic aneurysm/dissection | 441 | I71.x |  | •• |
| **Peripheral vascular disease (0-2)** | Diabetic PVD | 250.7, 249.7 | E08, E09, E10,  E11,E13 Relevant Subcodes E**.51, E**.59, E**.621 | • |  |
|  | Other aneurysm, LE | 442.3 | I72.4 | • |  |
|  | PVD | 440.21, 443.81, 443.9 | I70.21x I73.89 I73.9 | • |  |
|  | Foot wound + complication | 892.1 | S91.3x | • |  |
|  | Embolism/thrombosis (LE) | 444.22 | I74.3 |  | •• |
|  | Gangrene | 785.4 | E10.52, E11.52 I96 |  | •• |
|  | Gas gangrene | 040.0 | A48.0 |  | •• |
|  | Ulcer of lower limbs | 707.1 | L97.x |  | •• |
| **Metabolic (0-2)** | Ketoacidosis | 250.1, 249.1 | E1010, E1011 |  | •• |
|  | Hyperosmolar | 250.2, 249.2 | E1101, E1100 |  | •• |
|  | Other coma | 250.3, 249.3 | E10641, E11641 |  | •• |
| Reference: Glasheen WP, Renda A, Dong Y. Diabetes Complications Severity Index (DCSI)-Update and ICD-10 translation. *J Diabetes Complications*. 2017;31(6):1007-1013 | | | | | |

| **SUPPLEMENTAL TABLE 3. Factors associated with acute kidney injury occurrences.** | | | | | | | | | | |
| --- | --- | --- | --- | --- | --- | --- | --- | --- | --- | --- |
| Variables | | No. of patients | No. of  events | Person-time (years) | Adjusted HR | 95% CI | | | *p-value* |  |
| **Study cohort** | |  |  |  |  |  |  |  |  |  |
|  | Linagliptin group | 3521 | 144 | 5238.6 | 1.00 | (Reference) | | |  |  |
|  | Empagliflozin group | 3521 | 67 | 4687.3 | 0.60 | (0.45 | - | 0.82) | 0.001 |  |
| **Age group, years** | |  |  |  |  |  |  |  |  |  |
|  | < 65 | 4727 | 132 | 6729.4 | 1.00 | (Reference) | | |  |  |
|  | ≧65 | 2315 | 79 | 3196.5 | 0.87 | (0.64 | - | 1.19) | 0.38 |  |
| **Sex (Male vs. Female)** | | 4154 | 148 | 5838.8 | 1.37 | (1.01 | - | 1.86) | 0.05 |  |
| **eGFR (mL/min/1.73 m2)** | |  |  |  |  |  | | |  |  |
|  | 60-89.9 vs. ≧90 | 2895 | 95 | 4093.8 | 1.55 | (1.07 | - | 2.24) | 0.02 |  |
|  | 45-59.9 vs. ≧90 | 1114 | 63 | 1521.0 | 1.95 | (1.25 | - | 3.06) | 0.003 |  |
| **HbA1C** | |  |  |  |  |  |  |  |  |  |
|  | >7.0 vs. ≦7.0 | 5834 | 167 | 8250.8 | 1.03 | (0.70 | - | 1.51) | 0.88 |  |
|  | Missing vs. ≦ 7.0 | 111 | 4 | 134.6 | 0.58 | (0.19 | - | 1.80) | 0.35 |  |
| **UACR** | |  |  |  |  |  |  |  |  |  |
|  | No proteinuria | 2267 | 31 | 3288.9 | 1.00 | (Reference) | | |  |  |
|  | Microproteinuria | 1260 | 44 | 1769.7 | 2.22 | (1.36 | - | 3.62) | 0.002 |  |
|  | Macroproteinuria | 596 | 45 | 802.8 | 3.13 | (1.87 | - | 5.24) | <.001 |  |
|  | Missing | 2919 | 91 | 4064.5 | 2.05 | (1.33 | - | 3.17) | 0.001 |  |
| **Baseline Comorbidity** | |  |  |  |  |  |  |  |  |  |
|  | Retinopathy | 499 | 34 | 664.0 | 1.82 | (1.22 | - | 2.74) | 0.004 |  |
|  | Nephropathy | 1791 | 77 | 2559.8 | 0.93 | (0.65 | - | 1.34) | 0.71 |  |
|  | Neuropathy | 659 | 22 | 944.3 | 0.74 | (0.46 | - | 1.18) | 0.21 |  |
|  | Cerebrovascular | 492 | 22 | 659.9 | 1.42 | (0.88 | - | 2.30) | 0.15 |  |
|  | Angina pectoris | 533 | 19 | 767.4 | 0.79 | (0.47 | - | 1.32) | 0.36 |  |
|  | Myocardial infarction | 280 | 18 | 356.7 | 1.16 | (0.65 | - | 2.08) | 0.62 |  |
|  | Heart failure | 431 | 38 | 554.6 | 1.07 | (0.68 | - | 1.68) | 0.77 |  |
|  | Peripheral vascular disease | 199 | 9 | 273.2 | 1.08 | (0.53 | - | 2.21) | 0.83 |  |
|  | Metabolic | 13 | 2 | 18.7 | 1.78 | (0.36 | - | 8.73) | 0.48 |  |
|  | Hypertension | 4383 | 154 | 6170.8 | 1.29 | (0.88 | - | 1.87) | 0.19 |  |
|  | Hyperlipidemia | 4422 | 118 | 6368.7 | 0.96 | (0.68 | - | 1.36) | 0.83 |  |
|  | Peptic ulcer | 817 | 43 | 1147.5 | 1.67 | (1.16 | - | 2.41) | 0.006 |  |
|  | Liver diseases | 1085 | 40 | 1602.9 | 1.13 | (0.76 | - | 1.66) | 0.55 |  |
|  | Cancer | 465 | 43 | 591.6 | 2.24 | (1.51 | - | 3.34) | <.001 |  |
|  | Severe liver diseases | 18 | 2 | 21.5 | 0.43 | (0.08 | - | 2.29) | 0.33 |  |
|  | Metastatic cancer | 45 | 7 | 46.1 | 2.88 | (1.20 | - | 6.91) | 0.018 |  |

| **SUPPLEMENTAL TABLE 3. Factors associated with acute kidney injury occurrences (continued).** | | | | | | | | | | |
| --- | --- | --- | --- | --- | --- | --- | --- | --- | --- | --- |
| Variables | | | No. of patients | No. of  events | Person-time (years) | Adjusted HR | 95% CI | | | *p-value* |
| **Prior medication** | | |  |  |  |  |  |  |  |  |
|  | **Other antidiabetic agents** | |  |  |  |  |  |  |  |  |
|  |  | Insulins | 813 | 47 | 1128.6 | 1.31 | (0.90 | - | 1.91) | 0.16 |
|  |  | Metformin | 6807 | 196 | 9652.4 | 0.63 | (0.35 | - | 1.12) | 0.12 |
|  |  | Sulfonylureas | 4133 | 133 | 6007.8 | 1.02 | (0.75 | - | 1.39) | 0.91 |
|  |  | Acarbose | 2265 | 80 | 3343.5 | 1.14 | (0.83 | - | 1.57) | 0.41 |
|  |  | Thiazolidinediones | 1656 | 35 | 2466.4 | 0.57 | (0.38 | - | 0.85) | 0.006 |
|  |  | Glucagon-like peptide-1 | 386 | 15 | 561.1 | 1.23 | (0.70 | - | 2.17) | 0.47 |
|  |  | Meglitinides | 670 | 33 | 992.8 | 1.19 | (0.79 | - | 1.79) | 0.40 |
|  | **Lipid-lowering agents** | |  |  |  |  |  |  |  |  |
|  |  | Statins | 4290 | 119 | 6145.3 | 0.79 | (0.56 | - | 1.11) | 0.17 |
|  |  | Fibrates | 756 | 18 | 1102.3 | 0.76 | (0.45 | - | 1.27) | 0.29 |
|  |  | Others | 197 | 6 | 287.1 | 0.96 | (0.41 | - | 2.23) | 0.92 |
|  | **Anti-hypertensive medication** | |  |  |  |  |  |  |  |  |
|  |  | ACEI | 450 | 17 | 666.8 | 0.77 | (0.42 | - | 1.42) | 0.40 |
|  |  | ARB | 3659 | 114 | 5192.2 | 0.85 | (0.59 | - | 1.23) | 0.39 |
|  |  | Direct renin inhibitor | 20 | 2 | 30.2 | 3.91 | (0.93 | - | 16.54) | 0.06 |
|  |  | Thiazide | 154 | 10 | 230.2 | 1.22 | (0.57 | - | 2.65) | 0.61 |
|  |  | Furosemide | 362 | 39 | 461.7 | 0.80 | (0.51 | - | 1.25) | 0.33 |
|  |  | Diuretics-potassium sparing | 276 | 32 | 363.1 | 1.12 | (0.64 | - | 1.98) | 0.69 |
|  |  | Beta-blockers | 2186 | 88 | 3080.2 | 1.09 | (0.79 | - | 1.51) | 0.59 |
|  |  | Calcium channel blockers | 1530 | 66 | 2158.7 | 1.25 | (0.91 | - | 1.74) | 0.18 |
|  | **NSAID** | | 612 | 24 | 833.0 | 0.73 | (0.46 | - | 1.16) | 0.19 |
| **Concomitant medications** | | |  |  |  |  |  |  |  |  |
|  | NSAID | | 595 | 60 | 786.3 | 3.56 | (2.52 | - | 5.05) | <.001 |
|  | Antibacterial agents for system use | | 214 | 38 | 274.8 | 3.06 | (2.06 | - | 4.55) | <.001 |
|  | **Anti-hypertensive medication** | |  |  |  |  |  |  |  |  |
|  |  | Thiazide | 107 | 8 | 162.5 | 1.87 | (0.79 | - | 4.42) | 0.16 |
|  |  | Furosemide | 390 | 79 | 460.5 | 5.22 | (3.50 | - | 7.76) | <.001 |
|  |  | Diuretics-potassium sparing | 265 | 43 | 310.7 | 2.55 | (1.53 | - | 4.23) | <.001 |
|  |  | ACEI | 314 | 21 | 445.2 | 1.10 | (0.61 | - | 2.00) | 0.74 |
|  |  | ARB | 2883 | 85 | 4068.2 | 0.82 | (0.59 | - | 1.15) | 0.26 |
|  | Immunosuppressants | | 44 | 4 | 56.2 | 2.44 | (0.87 | - | 6.80) | 0.09 |
|  | Antiviral agents for system use | | 12 | 3 | 15.7 | 5.26 | (1.49 | - | 18.52) | 0.01 |
|  | Contrast media | | 17 | 6 | 21.3 | 6.90 | (2.74 | - | 17.37) | <.001 |

ACEIs: Angiotensin-converting-enzyme inhibitors; ARBs: Angiotensin II receptor blockers; eGFR: estimated glomerular

filtration rate; NSAID: non steroid anti-inflammatory drug; UACR: urine albumin-creatinine ratio

| **SUPPLEMENTAL TABLE 4. Linear mixed model for the decline changes in eGFRs.^+^** | | | | | | |
| --- | --- | --- | --- | --- | --- | --- |
| Variables | | Adjusted coefficient (β) | 95% CI | | | *P* value |
| **Treatment group** | |  |  |  |  |  |
|  | Linagliptin | Reference | | | |  |
|  | Empagliflozin | 1.51 | (0.30 | - | 2.72) | 0.01 |
| **Follow-up time (in quarter)*** | | -0.72 | (-0.81 | - | -0.64) | <.001 |
| **Study cohort*Follow-up time** | |  |  |  |  |  |
|  | Linagliptin*time | Reference | | | |  |
|  | Empagliflozin*time | 0.27 | (0.15 | - | 0.40) | <.001 |
| **Acute kidney injury during follow-up time** | |  |  |  |  |  |
|  | No | Reference | | | |  |
|  | Yes | -11.89 | (-15.36 | - | -8.42) | <.001 |
| **Age group, years** | |  |  |  |  |  |
|  | <65 | Reference | | | |  |
|  | ≧65 | -17.31 | (-18.57 | - | -16.05) | <.001 |
| **Sex** | |  |  |  |  |  |
|  | Female | Reference | | | |  |
|  | Male | -9.47 | (-10.67 | - | -8.27) | <.001 |
| **Baseline HbA1C** | |  |  |  |  |  |
|  | ≦7.0 | Reference | | | |  |
|  | > 7.0 | 2.88 | (1.25 | - | 4.52) | 0.001 |
|  | Missing | 1.76 | (-3.19 | - | 6.71) | 0.49 |
| ^+^ eGFR: estimated glomerular filtration rate *Follow-up time was the number of eGFR measurement in a 3-month interval over the study period. | | | | | | |

**SUPPLEMENTAL FIGURE 1.** Estimated glomerular filtration rate (eGFR) trajectory between empagliflozin and linagliptin groups**.** A. PSM cohort (N=7042); B. patient with acute kidney injury (N=211); C. patients without acute kidney injury (N=6831).


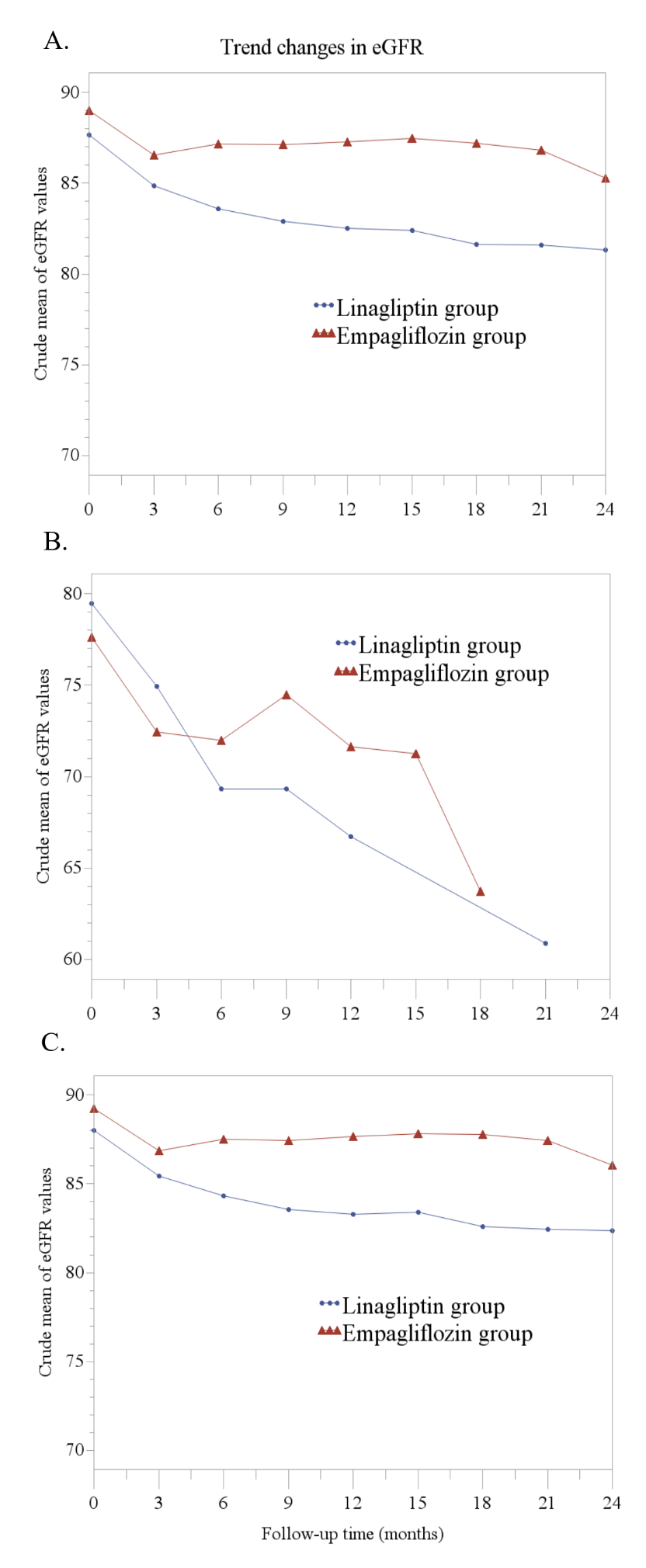

Supplement: Supplementary file 1 [file DataSheet1.docx]
